# ﻿Lectotypification and nomenclature notes of the name *Caraganaopulens* (Fabaceae, Papilionoideae) and its synonyms

**DOI:** 10.3897/phytokeys.226.104110

**Published:** 2023-05-12

**Authors:** Shabir A. Rather, Anand Kumar, Hongmei Liu

**Affiliations:** 1 Center for Integrative Conservation & Yunnan Key Laboratory for Conservation of Tropical Rainforests and Asian Elephants, Xishuangbanna Tropical Botanical Garden, Chinese Academy of Sciences, Mengla, Menglun 666303, Yunnan, China Xishuangbanna Tropical Botanical Garden, Chinese Academy of Sciences Mengla China; 2 Central National Herbarium, Botanical Survey of India, P.O. Botanic Garden, Howrah-711 103, West Bengal, India Central National Herbarium, Botanical Survey of India, Howrah India

**Keywords:** Lectotype, Leguminosae, opulens complex, overlapping traits, taxonomy

## Abstract

Morphological characters currently used to differentiate *Caraganaopulens* as a species have been found to be insufficient and inconsistent. Through extensive research and comparisons of specimens, it has been revealed that *C.opulens* and its synonyms have overlapping geographical distributions, and that typification is necessary for *C.opulens*. Therefore, a lectotype is designated for the name *C.opulens*, with comments on its typification. Additionally, the current typification status is discussed for all its synonyms, accompanied by substantive notes.

## ﻿Introduction

The legume genus *Caragana* Fabr. (Fabaceae, Papilionoideae) is ecologically and pharmacologically important, comprising approximately 100 species distributed in temperate arid and semi-arid areas of the Northern Hemisphere (Polhill 1981; Zhang et al. 2009; Rather et al. 2021). China alone hosts more than 70% of the species diversity of these legumes, with about 66 species (Li and Ni 1985). The Euro-Asian range, which includes 80 species, extends towards Japan, Korea, and Siberia in the north and northeast, towards Central Asia and Europe in the west, and along the Himalaya towards Northern India, Bhutan, and Nepal in the south (Polhill 1981; Lock 2005; Liu et al. 2010; Rather et al. 2021).

Identifying closely related species in *Caragana* has been challenging through the alpha taxonomic approach, as some species have overlapping morphological traits. One such case is of the *C.opulens* species complex, consisting of three species, namely *C.opulens*, *C.kansuensis* and *C.licentiana*. Although diagnostic characters have been identified, such as ovary/fruit pubescence, leaf shape, bract shape, and leaf pubescence, these traits have been found to be variable and labile (Moore et al. 2010). To make matters worse, several specimens housed in major herbaria in China have been misidentified (Zhao 1993). Extensive investigation of literature and comparison of specimens has revealed that these species have overlapping geographical distribution and require lectotypification.

During our ongoing revisionary studies of the genus *Caragana* in the Pan-Himalaya region, we found the typification of *C.opulens* Kom. requires clarification by designating a lectotype. By carefully studying the protologue and the material arguably studied by Komarov, we designated the lectotype for the above name. Furthermore, we discussed the types of all synonyms of this species, including *C.kansuensis* Pojark., *C.licentiana* Hand.-Mazz., C.opulensvar.angustifolia Y.Z. Zhao ex Zhao Y. Chang & F.C. Shi., C.opulensvar.perforata Merrgen & Ma., and C.opulensvar.trichophylla Z.H. Gao & S.C. Zhang. Nomenclatural notes discussing the selection of type specimens are given for each name, and known isotypes are also cited.

## ﻿Material and method

This study is based on the examination of relevant literature on the floristics of China and adjacent nations, and the examination of specimens held in the following herbaria: LE, P, PE, and W (Brach and Song 2006). We made special efforts to examine material studied by Komorov by reaching out to the curators of the Komarov Botanical Institute (LE) and the Naturhistorisches Museum Wien (W) to inquire about the availability of type specimens. To select types, we compared images and specimens with protologues, and the most complete and representative specimens were selected as lectotype for the name, according to Art. 9.3 of the “Shenzhen Code” (Turland et al. 2018).

## ﻿Taxonomic treatment

### ﻿Types of *Caraganaopulens* and its synonyms

#### ﻿ Caragana opulens

Taxon classificationPlantaeFabalesFabaceae

Kom., Trudy Imp. S.-Peterburgsk. Bot. Sada. 29: 208. 1908.

5CB27E89-D0AD-5454-A0E5-5750F32992E0

##### Type.

**Figure 1.**
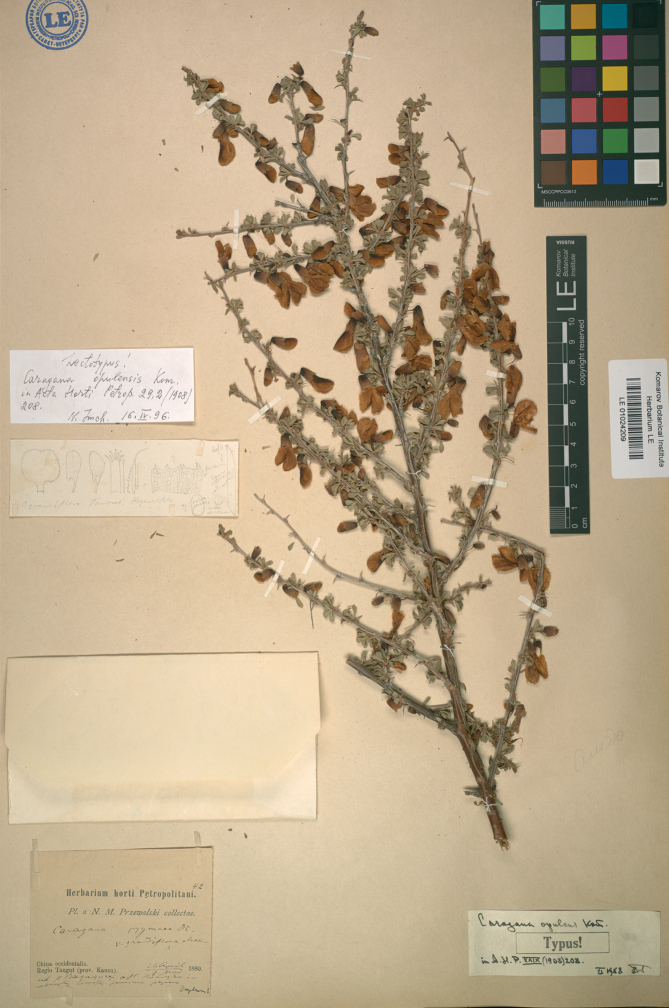
Lectotype of *Caraganaopulens* Kom. (LE01024209). Komarov Botanical Institute, St. Petersburg, Russia (LE).

China. Kansu: Bara-Topra, 26 April 1880, *N.M. Przewalski 42* (***lectotype*** LE [barcode LE01024209, image!], designated here, Fig. 1). Remaining syntypes: China. Kansu: Bara-Topra, 11 May 1880, *N.M. Przewalski s.n.* (LE [barcode LE01024210, image!]). China. Thibet [Tibet]: route de Lhassa à Batang, May 1890, *Henry D’Orléans s.n.* (P [barcode P02767097, image!]).

#### ﻿ Caragana licentiana

Taxon classificationPlantaeFabalesFabaceae

Hand.-Mazz., Oesterr. Bot. Z. 82: 249. 1933.

A8A166A5-013C-519C-8D5C-FC442CE754AE

##### Type.

**Figure 2.**
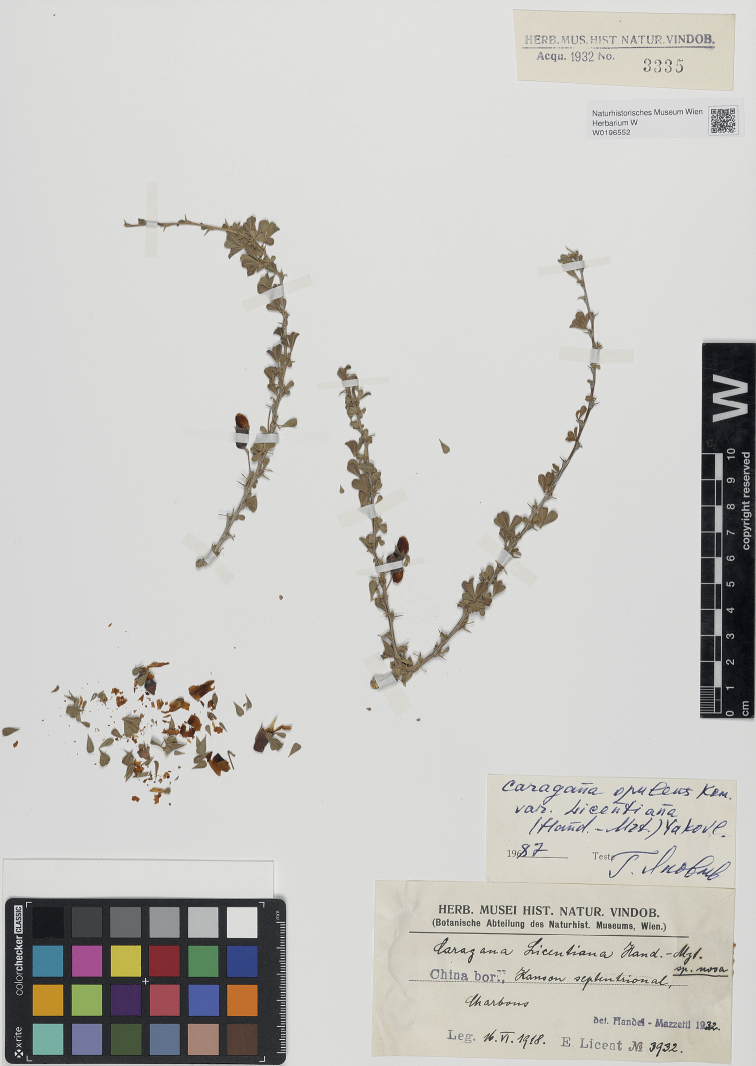
Holotype of *Caraganalicentiana* Hand.-Mazz. (W0196552). Natural History Museum, Vienna-Herbarium (W).

China. Kansu, 16 June 1918, *Licent 3932* (***holotype*** W [barcode W0196552, image!, Fig. 2]; isotypes K [barcode K000511783, image!], P [barcode P02767130, image!]). China. Kansu: Kiangra, 9 October 1918, *Licent 4908* (paratypes W [barcode W0016458, image!], P [barcode P02767129, image!]).

#### ﻿ Caragana kansuensis

Taxon classificationPlantaeFabalesFabaceae

Pojark., Bot. Mater. Gerb. Bot. Inst. Komarova Akad. Nauk S.S.S.R. 13: 138. 1950.

B1D66076-42A0-515F-92AB-78A353565342

##### Type.

**Figure 3.**
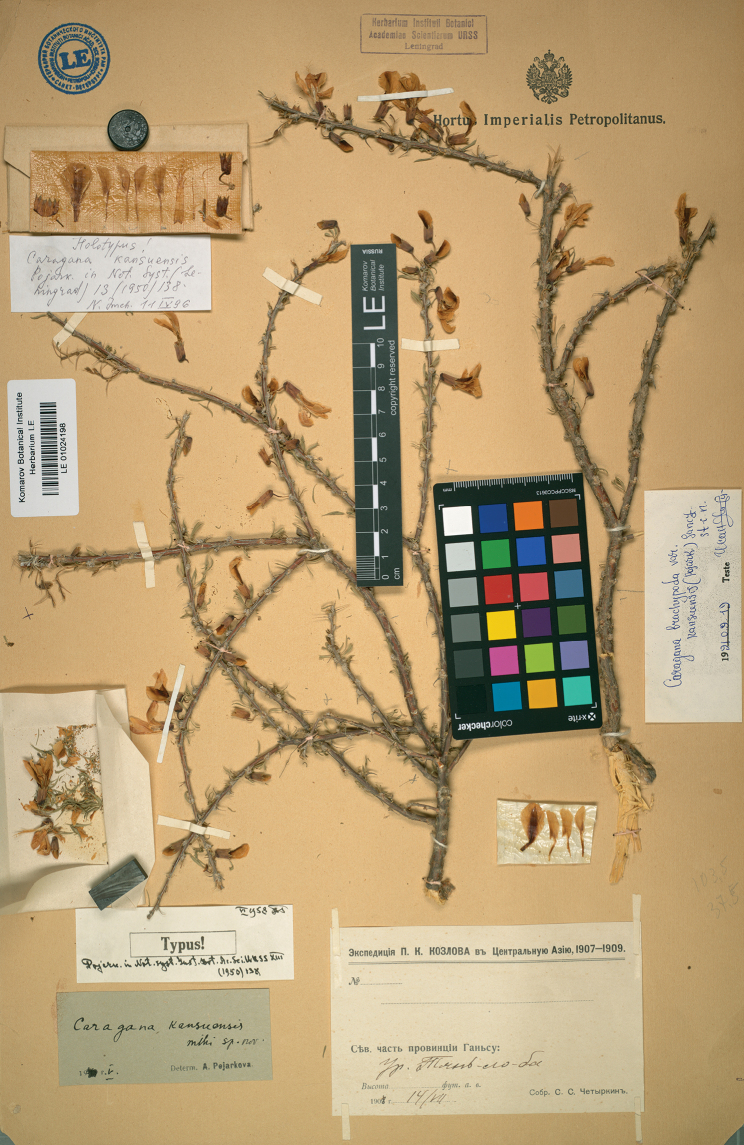
Holotype of *Caraganakansuensis* Pojark. (LE01024198) Komarov Botanical Institute, St. Petersburg, Russia (LE).

China. Kansu [Gansu]: Tjan-lo-ba dicto, 14 July 1908, *S. Czetyrkin s.n.* (***holotype*** LE [barcode LE01024198, image!, Fig. 3]).

#### ﻿ Caragana opulens var. perforata

Taxon classificationPlantaeFabalesFabaceae

Merrgen & Ma, Acta Sci. Nat. Univ. Intramongol. 20: 554. 1989.

69754938-7357-55B3-A7E7-E2307439BB29

##### Type.

China. Nei Mongol: Hohhot, Daqing Shan, 31 May 1973, *Y.C. Ma & C.J. Wu 32* (***holotype*** HIMC [barcode HIMC0017878, image!] [https://www.cvh.ac.cn/spms/detail.php?id=e33a1be3]). ***Paratypes***: China. Nei Mongol: Baotou, Wudang Zhao, 8 July 1959, *Y.C. Ma et al. 4-47* (HIMC [barcode HIMC0017877, image!]). China. Ulanqab Meng, Helinger, 19 July 1959, *Y.C. Ma et al. 1-173* (HIMC [barcode HIMC0017879, image!]).

#### ﻿ Caragana opulens var. trichophylla

Taxon classificationPlantaeFabalesFabaceae

Z.H. Gao & S.C. Zhang, Bull. Bot. Res., Harbin 9(3): 63. 1989.

FA9A26CE-C94F-569D-8BE6-3EA228C94AB7

##### Type.

China. Gansu: Minqinxian (Cultivated in Garden of Minqin Desert Botanica), 13 May 1988, *Z.H. Gao & S.C. Zhang 88001* (MQ, *n.v.*). Paratype: China. Gansu: Qilianshan, Qilianlinchang, *Z.H. Gao 86002* (MQ, *n.v.*).

#### ﻿ Caragana opulens var. angustifolia

Taxon classificationPlantaeFabalesFabaceae

Y.Z. Zhao ex Zhao Y. Chang & F.C. Shi, Bull. Bot. Res., Harbin 31: 136. 2011.

7C00A7A1-A819-5196-9278-20264C1E42A0

##### Type.

China. Shaanxi: Suide, Jiuyuangou, near Wujiapan, 20 May 1956, *Huanghe Exped. 6879* (***holotype*** WUK [barcode WUK0086537, image!] [https://www.cvh.ac.cn/spms/detail.php?id=bfd87e11]; isotype PE [barcode PE00180919, image!]). ***Paratypes***: China. Shanxi: Wuzhai, Hanjialou, on a slope, alt. 1400 m, 13 June 1965, *J.X. Yang 3303* (WUK [barcode WUK024889, image!], HNWP [barcode HNWP86481, image!]). China. Shanxi province: Xingxian, Caijiaya, on a slope, 22 August 1955, *Huanghe Exped. 2074* (PE [barcode PE01580673, image!]) China. Shaanxi: Yanchuan, near Suyahe, alt. 580 m, 5 September 1955, *K.T. Fu 7857* (WUK, *n.v.*).

### ﻿Nomenclature notes

*Caraganaopulens* was described by the famous Russian botanist Vladimir Leontyevich Komarov (1869–1945) in 1908, who cited accessions from three major provinces: Gansu, Mongolia, and Tibet (Komarov 1908). The protologue included a total of eleven accessions, including four syntypes from Mongolia, six from Gansu, and one from Tibet. We were able to retrieve three specimens, namely two specimens at LE and one at P. Additionally, we found one specimen at PE with a label indicating ‘Paratypus’, but it lacked any herbarium collection label. Most importantly, there was no annotation by Komarov. Thus, no evidence supported that Komarov studied this specimen when he described *C.opulens*. Therefore, we excluded this specimen from our study. As Komarov worked at St. Peterburg, many accessions studied by him are expected to be held at the institute now named after him, the Komarov Botanical Institute at St. Petersburg, Russia (LE). Therefore, we gave preference to designate a lectotype from the collection maintained at LE. Two syntype specimens were present at LE, but the specimen was collected by the Russian explorer N.M. Przewalski (1839–1888) from Gansu, with the barcode number LE01024209, was the closest match to the protologue. Additionally, the specimen also bore pencil line drawings of dissected parts of flowers and fruit. Although the specimen bore the annotation ‘Lectotypus’ by N. Imch, this designation was never published. Here, we proposed to accept N. Imch’s selection and designated this specimen as the lectotype of *Caraganaopulens*, according to Art. 9.3 of the “Shenzhen Code” (Turland et al. 2018).

In 1933, the Austrian botanist Heinrich Raphael Eduard Handel-Mazzetti (1882–1940) described *Caraganalicentiana* Hand.-Mazz. based on two collections, *Licent 3932* and *Licent 4908*, from the Gansu Province of China (Handel-Mazzetti 1933). In the protologue, *Licent 3932* was cited as the type. Furthermore, Handel-Mazzetti (1931) stated, “Die Exemplare befinden sich im Herbar des Naturhistorischen Museums in Wien” [The specimens are in the Herbarium of the Natural History Museum in Vienna]. We corresponded with the curator of the W and received one image of *Licent 3932* and one image of *Licent 4908*. Thus, the holotype *Licent 3932* was confirmed to be housed at W, while the duplicates at K and P represented isotypes. *Licent 4908* housed at W and P were assigned to be paratypes.

The name *Caraganakansuensis* Pojark. was described based on a collection by S. Czetyrkin obtained from the Gansu Province of China (Pojarkova 1950). We traced a single specimen of S. Czetyrkin at LE bearing Pojarkova’s annotation ‘*Caraganakansuensis* sp. nov.’ in her own handwriting. The specimen also comprised the dissected parts of flowers. Each and every part of the drawing of the protologue was also prepared from this specimen by the author. Therefore, we found conclusive evidence that this specimen represented the holotype.

## Supplementary Material

XML Treatment for Caragana opulens

XML Treatment for Caragana licentiana

XML Treatment for Caragana kansuensis

XML Treatment for Caragana opulens var. perforata

XML Treatment for Caragana opulens var. trichophylla

XML Treatment for Caragana opulens var. angustifolia
